# An Analysis of Fourcroy's Observations on the Experiments Made by the English Chemist Mayow, towards the End of the Seventeenth Century

**Published:** 1799-05

**Authors:** 


### *An Analysis of* Fourcroy's *Observations on the Experiments made by the English Chemist* Mayow, *towards the End of the seventeenth Century.*

[Extracted from the 'Annales de Chimie,' No. 85.]

THE author begins with observing, that Mayow's discoveries were not made known to the world, till his works were reprinted in 1790, in England; that Lavoisier took no notice of this chemist, who without dispute preceded Boyle and Hales in the path generally believed to have been opened by these illustrious philosophers; and that Mayow had even proceeded farther, and more decisively, than his predecessors before mentioned. Mayow's work seems to present a variety of truths and discoveries brought forward by the chemists of the present day. The correspondence of his ideas, with the present pneumatic doctrine, is striking. It is therefore just, says Fourcroy, to restore to the memory of this physician what is due to him; but it is also just to point out the difference between the discoveries, and even the experiments of Mayow, ingenious and conclusive as they appear, and the exact researches of the philosophers of our day, as well as the certain results drawn from thence.

John Mayow, physician in London, published at Oxford, in 1669, a work containing five Latin Dissertations, which were reprinted together in 1674, and 1681, under the title—"*Johannis Mayow, &c. opera omnia medico-physica, tractatibus quinque comprehensa.*" Two of these treatises, the one *On saltpetre and the nitro-aërial spirit*, and the other *On respiration*, contain a number of singular and new ideas, on the agency of the air in combustion and respiration; on its diminution and absorption by these two phenomena; on the analogy subsisting between air and nitre in supporting inflammation; on the identity of the principle of air with the acid of nitre, and on the formation of that acid by the igneo-aërial spirit. We find here asserted, in the clearest and most positive manner, that the blood absorbs a part of the air called vital; that the blood is heated by that absorption, or by the presence of the air in the lungs. Mayow also demonstrates, that the red colour of blood, and the change of venous into arterial blood, depend entirely on its contact with the atmospheric air. In a word, this work contains a number of ideas, which have now become demonstrated truths, since natural philosophy and chemistry have determined the real influence of the air in combustion, in the calcination of metals, and the formation of acids; and if Mayow had been able to exhibit, in a separate state, the vital air of the atmosphere, and extract the substances therein absorbed, or held in solution; if he had known its eſſential properties, and deſcribed them in a clear and diſtinct manner, he would have eſtabliſhed the whole baſis of the modern theory of combuſtion; the formation of acids; and of reſpiration;—of which he has, however, given only general data.

The point of view in which Fourcroy propoſes to conſider this ſubject, is to preſent a ſucceſſion of machines, apparatus, and medico-chemical proceſſes, by means of which Mayow endeavoured to aſcertain, by experiments, the action of inflammable bodies, and the reſpiration of animals on the air, as well as the reciprocal influence of the air on theſe phenomena: taking at the ſame time a view of the principal theories of that author.

In examining the fifth plate of the work cited, and which is placed at the end of the fourth treatiſe, '*De motu muſculari*,' oppoſite to page 383 of the Hague octavo edition, we are aſtoniſhed to recognize, at once, the apparatus repreſented in ſix figures to be nearly the ſame, not only with thoſe which Hales uſed, and had engraved, fifty years poſterior to this philoſopher, without taking notice of, and perhaps even without being acquainted with thoſe of Mayow, but alſo with the machines of Prieſtley and Lavoiſier; contrivances which have been ſuppoſed to be perfectly new, a century after the diſcovery of the Engliſh phyſician. By theſe, particularly the glaſs retorts, he was able to determine, that air was in part fixed, or diminiſhed and abſorbed by combuſtion and reſpiration; that theſe two phenomena were attended with a ſimilar effect; that this property of promoting the one or the other was peculiar to the air; in a word, that there was only one part of the air, namely, the moſt ſubtle and elaſtic, that could be called vital, and which ſerved to promote inflammation and reſpiration.

Mayow's firſt and moſt important diſſertation principally aims at explaining the nature of nitre; its ſpontaneous production; the analogy of its acid with the air; the exiſtence of a principle in the atmoſphere, of the ſame nature with nitre, which ſupports combuſtion, flame, and life; a very active principle in all chemical phenomena, and in the properties of which we cannot but ſee the moſt ſtriking correſpondence with the vital air, or oxygenous gas, diſcovered by Prieſtley, a hundred years after.

In the ſeventh, eighth, and ninth chapters of the firſt treatiſe, Mayow deſcribes the experiments by which he ſupports his theory of combuſtion and reſpiration, after having premiſed, that the air contains particles which he calls *nitro-aërial*, and which are neceſſary to produce fire; ſo that when that element is once deprived of theſe, the air can no longer renew inflammation; and, according to the degree in which it is thus deprived, it loſes its form and elaſticity;—he ſhews, that the flame being extinguiſhed in a cuppingcupping-glafs applied to the fkin, it caufed a vacuum; the fkin, preffed by the external air, became prominent within the glafs; and this vacuum, as well as the extinction of the flame, proceeds from the circumftance, that the nitro-aërial part was abforbed, and had loft its elafticity. To fhew the manner in which Mayow proves this affertion, Fourcroy cites a variety of experiments from the feventh chapter of this differtation, which, he obferves, appear to prove, that Mayow perfectly underftood the influence of the air in refpiration; that he had formed a conjecture of the nature of the atmofphere; that he conceived the abforption and true diminution of its vital part, by combuftion and refpiration; that, in a word, thefe paffages would evince a glorious priority of difcovery in Mayow; but from the fubfequent paffages it appears, that although he had proceeded to a certain length, in that courfe in which he had no competitor or fucceffor for upwards of fifty years, yet that he did not go far enough, nor purfue his progrefs with that firmnefs which was neceffary to afcertain and eftablifh the truth.

In the 8th chapter, proceeds Fourcroy, Mayow fpeaks of the nitro-aërial fpirit as abforbed by animals, "*De fpiritu nitro-aëreo quatenus ab animalibus hauritur*", and treats, at confiderable length, of the phenomena of refpiration, with refpect to the air. He had, he fays, in a former work, proved that the principal ufe of that function was to feparate the air, and to unite to the mafs of the blood, by means of the lungs, certain particles neceffary to the fupport of life; that the air proceeding from the lungs of animals, is deprived of certain elaftic parts, and its volume is therefore confiderably diminifhed, in proportion to the abftraction of the nitro-aërial particles. To defcribe the manner of that feparation, and to prove that it is caufed by penetrating into the mafs of the blood, and depofiting there fuch particles, he enumerates fome experiments, which our limits will not permit us to detail, but which corroborate the evidence of his having arrived at the knowledge of the nitrous air or gas, without being fenfible of the difcovery; but here again there appears the fame kind of diffidence and confufed reafoning, which every where fhews this ingenious difcoverer to have been, in many refpects, irrefolute, and not fully mafter of the grounds on which he proceeded; and ftill we read of the lofs of elafticity of the nitro-aërial fpirit, with a diminution of volume: he has, however, been aware, that vapour, difengaged by the effervefcence of the fpirit of nitre with iron, did not proceed from the air, which was diminifhed only in proportion as the nitro-aërial fpirit was exhaufted. His intention is to explain, how that fpirit is condenfed by the blood, and to this end he directs the refult of his experiments. He compares blood to a mixture of iron and acid, acid, and ftates them to be liable to a fimilar fermentation; and as he explains the condenfation of the nitro-aërial fpirit by this fermentation, it is pretty evident, that to fuch fermentation of the blood—he attributes the fame condenfation in the procefs of refpiration. Here, fays Fourcroy, he feems to have got into falfe reafoning:—the fermentation of the blood proceeds from the condenfation of the nitro-aërial fpirit, and that condenfation is produced by the fermentation of the blood;—the particles of that fpirit being once condenfed, he admits their introduction into the mafs of the blood, and makes them at the fame time very powerful agents. He attributes to them the red colour, the heat, and fluidity of the blood. From this caufe he believes, that the part of the blood is black, which is not in contact with the air, and of a florid colour in the contrary cafe; that the venous blood is black, and the arterial remarkably red; that violent exercife, as it accelerates infpiration, is overheating, as is alfo frequent voluntary refpiration; that the fever of the confumptive proceeds from the force with which the morbid matter in the lungs attracts and abforbs the the nitro-aërial fpirit, with which it enters into a ftrong fermentation; that if the venous nnd arterial blood be placed in the vacuum of Boyle, the former prefents only fome globules, and the latter is entirely diffipated in froth. He further adds, that the nitro-aërial fpirit gives the red colour to bodies in which it exifts, not unlike the fuming fpirit of nitre. He compares the heat produced by the condenfation of this fpirit in the lungs, to that generated by a flint eliciting fparks, and alfo attributes vitriolifation to the fame caufe.

Fourcroy proceeds to the ninth chapter of Mayow, and details from thence a variety of proceffes to convey the air or elaftic emanation from one jar into another, or to admit a part of it, to transfer it from one veffel into another, to place them in a pneumatic receiver, in order to meafure the dilatation caufed by the vacuum; which is evidently the fame with the procefs of this day, or that of Hales, and fuperior to that of Boyle.

This ninth chapter is an inquiry into the production of air. Mayow was not fatisfied with difcovering the elafticity of the vapour he attracted, by the effervefcence of the fpirit of nitre with iron; he was further defirous to determine the analogy and difference of that vapour in proportion to common air. In this refpect, the apparatus he employed enabled him to extract, collect, and convey elaftic fluids; proceffes which have been fince loft for above a century, and are only reftored to us within thefe twenty years.

This article having been extended fo far, we cannot go into a detail of the feveral apparatus and proceffes employed by our countryman in his difcoveries veries. For thefe we muft refer the reader to his works, and fhall conclude by extracting Fourcroy's general obfervations, with which he accompanies his memoir.

"No philofopher, efpecially at the end of the laft century, had treated of the theory of combuftion and refpiration, the comparifon of thefe two natural phenomena, the reciprocal influence between them and the air, and the effects of that fluid, compared with thofe of nitre, on combuftible bodies, at fuch length and fo acutely as this Englifh phyfician: no perfon had contrived fo ingenious an apparatus and proceffes, altogether different from thofe employed by the philofophers of that day; nor made experiments fo novel to difcover the caufe of the neceffity of air in combuftion, as well as in refpiration; and, although neglected and unknown for more than a century, even condemned by his cotemporaries as altogether futile, thefe pneumatic proceffes undoubtedly deferved to be refcued from the oblivion in which they have been buried, for one hundred and twenty five years. But, while we render every juftice to Mayow, and his ingenious difcoveries, fome of which were far fuperior to the ideas of the moft celebrated philofophers of that age, and much refemble the purfuits and acquirements of thofe of the prefent day; while I feel pleafure in avenging his memory of the inconceivable contempt, or at leaft indifference, fhewn by his cotemporaries to his brilliant refearches, even among his compatriots, who are ufually jealous of the glory of their country: I muft fay, that he did not pufh, fo far as he might have done, the firft data which prefented themfelves to him; that the thread of his difcoveries was broken in his hands, and that he had little more than entered upon a career, of the extent of which he had no conception; that he was not even fufficiently ftruck with the novelty and importance of his firft difcoveries; that, inftead of purfuing the path of experiment which his new proceffes pointed out, he gave himfelf up to hypothetical reafonings, which interrupted his progrefs, and threw him into an ocean of uncertainties and contradictions, in obliging him fucceffively to admit and reject the condenfation, diminution, abforption, and fixation of air; in a word, that while he feems to difpute with the firft modern chemifts the invention of the pneumatic chemical apparatus, he leaves them, at the fame time, all the glory, and detracts nothing from their merits.

